# Hospital and Institutional News

**Published:** 1914-05-30

**Authors:** 


### HOSPITAL AND INSTITUTIONAL NEWS.

### OUR HOSPITAL SUNDAY APPEAL NUMBER.

This year, as usual, we shall issue a Special Appeal Number of The Hospital, which will be distributed to all the churches of London on the Sunday before Hospital Sunday—which falls this year on June 14. The object of this Special Number has always been to describe exhaustively the needs and claims of the voluntary hospitals in London, and in addition to the individual summary of the requirements of each institution, to set out what Hospital Sunday stands for in the life of the nation, and what the voluntary system represents as an expression of the national character. Attractive as well as authoritative articles by the clergy, the medical profession, and specially qualified laymen have always been made a feature, so that the general public, in the persons of all those who attend a place of worship on Hospital Sunday, may have brought to them in an authoritative, and, at the same time, an attractive way why and for what reasons the voluntary hospitals must be supported. Our task this year will be made considerably easier, and the interest of our readers be correspondingly enhanced by the special contribution which we hope to publish next week in the Special Appeal Number from Dr. Joseph Herman Hertz, the Chief Rabbi. The general public will need no reminder how large a part has always been taken by Jews in hospital work and in charity generally, and consequently no one could be more fitting to support the appeal of Hospital Sunday than the time-honoured official head of that community for which many of our hospitals provide special wards, and which as a whole gives and receives so much to and from the voluntary hospitals. In addition we hope to publish a special article dealing with the responsibility of the clergy to the Hospital Sunday Fund, and the difficulties which beset them in carrying it out. Arrangements have also been made to publish an article dealing with the Finance and Prospects of the Sunday Fund at the present time. Any reader of The Hospital can obtain the Special Number, price one penny, from The Scientific Press, 28 Southampton Street, Strand, W.C.

### STILL AMONGST THE ROCKS.

St. George's Hospital is not yet out of its difficulties, and the resignation of the matron, Miss McCall Anderson, and of the assistant matron, Miss Watson, are to be deeply regretted, for both these ladies have shown marked ability in their work at St. George's Hospital. The truth is that members of the honorary medical staff have been in a majority on the house committee for a considerable period. Until this grave novelty in the administrative arrangements of St. George's Hospital is effectually and finally removed, the best interests of the honorary medical staff and of the governors, together with the wise and businesslike management of this institution, must suffer. One serious danger lies in the direction of filling up vacancies on the resident staff. No great hospital can be efficient in its internal arrangements unless the principal residents are not only not habitually indolent, but invariably keen, active, tactful, untiring workers, ever anxious to promote and maintain efficiency throughout the establishment.

### CHANGES AT NORTH EVINGTON INFIRMARY.

The latest proposals of the Leicester Board of Guardians with regard to the medical staff of the North Evington Infirmary are an advance upon the proposals which we were compelled, at the beginning of this year, to criticise as inadequate. It is now suggested that the medical superintendent of the infirmary shall also act as medical officer of the workhouse at a salary of £520 per annum, rising to £570, with emoluments valued at £80. This is an increase of £120 per annum, and raises the salary to the amount we suggested was necessary in order to attract well-qualified candidates. Instead of one assistant medical officer at £150, as originally suggested, it has now been decided to advertise for two—one at £200 and the other at £150. We are glad to note that our criticisms have had this effect. The changes will make for greater efficiency, and that, in the long run, means greater economy. Our Report on the infirmary appears on page 237.

### STAFF RESIGNATIONS AT KING EDWARD VII.'S HOSPITAL, CARDIFF.

At a joint meeting of the chairmen of the various committees of the above hospital, held this week, it was decided, we understand, to accept the resignations of the three house surgeons at present in residence. The names of the three house surgeons are: Mr. J. F. Hill, M.B., B.Ch.; Mr. J. Prosser Davies, M.B., B.S.; and Mr. P. W. Ransom, M.B., B.C., M.R.C.S., L.R.C.P. Differences had arisen owing to a recent increase in the salary of the house physician, without similar recognition of the house surgeons. Although occupying a similar status in the hospital so we understand, the house physician is now paid £40 per annum more than the house surgeons.

### POOR-LAW INFIRMARIES AND INSURANCE CERTIFICATES.

The weekly certificates required under the Insurance Act in order that sick benefit may be drawn have thrown a great deal of additional work upon the medical officers of Poor-Law infirmaries. The official form of certificate requires that the patient shall be examined on the day it is signed, and that the nature of the illness shall be stated. Where, as in the case of large infirmaries, a number of such certificates has to be signed every day this takes up an appreciable amount of time. In Glasgow a conference has recently been held between representatives of the local Medical Committee, the Panel Committee, Medical institutions, and the Insurance Committee, with a view to securing coordination in the certification of insured persons for sickness benefit while under treatment in institutions. After examining the certificates in use in various local institutions, the conference decided to recommend all institutions to adopt the forms in use at the Royal and Western Infirmaries. These certificates are as follows: " This is to certify that . . . is at present an . . . patient of this Infirmary." They obviously meet all the requirements of the societies, and need much less time to fill in than the official forms. There seems to be no reason why a similar certificate should not be used universally in institutions. It may be noted, however, that a similar saving of time is obtained, if the official form is used, if the word " to-day " is deleted from the phrase " I have examined you to-day," and if the space for entering the nature of the disease is left blank. All approved societies appear to accept certificates from institutions filled up in this way, and the necessity for having special forms printed is avoided.

### A HISTORY OF BEDLAM.

A new institutional history is announced by Mr. Fisher Unwin for publication shortly. This is " The Story of Bethlehem Hospital," written by the Chaplain of that ancient institution, the Rev. E. G. O'Donoghue. The author has derived his materials from the archives of the hospital, and it is understood that he will present its history in full from the earliest foundation down to the present day. Bethlehem Hospital was founded nearly seven hundred years ago on a site somewhere close to Liverpool Street as a Priory of the Convent of Bethlehem. A century later it first became an institution for " lunatics," after moving to the site of Trafalgar Square. With such a splendid wealth of subject-matter the learned author should be able to make a most fascinating volume, and is to be congratulated upon his life's work in taking up the task. We shall hope to have an opportunity of reviewing this interesting work when it is published, and are glad to note this forthcoming addition to the somewhat slender list of institutional histories. " Bedlam " is worthy of a monograph, and it is very fitting that it should have one from the hand of its most competent authority.

### HEAD OF THE LONDON AMBULANCE SERVICE.

The London County Council propose to advertise without delay for applications for the position of a principal assistant in the Fire Brigade at a salary of £400 a year, rising by annual increments of £25 to £500 a year. This officer will be directly responsible for the discipline and effective working of the Ambulance Service. Candidates must be not less than thirty years of age and not more than forty-five, and members of the staff will not be precluded from making applications for the position. It has been decided that the body of the motor ambulances shall be painted white, with the inscription " L.C.C. Ambulance " on each side in red, and that an illuminated sign with " Ambulance " thereon shall be fixed in front of the canopy of each.

### THE DILKE MEMORIAL SCHEME.

We have recorded from time to time the progress, or, as impatient people would say, the want of progress, in the realisation of the scheme for giving a Dilke Memorial Hospital to the Forest of Dean. The late Sir Charles Dilke was formerly member of Parliament for this division, and the proposal in the first place was taken up with some enthusiasm. Interest has now flagged, and one might attribute this to the comparatively large sum of £6,000 which had been decided on, were it not for the fact, lately quoted at a meeting of the Dean Forest miners at Lydney by Mr. G. H. Rowlinson, the miners' agent, that one-half of this sum had been privately promised as soon as the other half has been subscribed. At this meeting Mr. Rowlinson pointed out that so far £1,600 had been raised, and followed this up with the practical proposal that a sum of £725 belonging to the miners—which had been originally subscribed for a local scheme that had been formed when they had contracted out of the Compensation Act, but had been lying idle since they had come under its amended provisions —should be voted to the hospital. He further pointed out that if they did not use it it would go to the Treasury, and so far carried his audience with him that his proposal was carried with practical unanimity. As a sum of £150 is understood to be available from another source, the position really comes to this, that only a further £500 is needed to produce the £3,000, which will instantaneously double itself, thanks to the generosity of the private donor alluded to. Of the £6,000 a proportion has been ear-marked for endowment, so it seems as if a special effort is all that is needed to make the Dilke Memorial Hospital scheme a financial if not immediately an architectural fact. A site and £25 a year have even been offered by the Office of Woods. Let the locality also remember this, that while every case must be judged on its merits, it is broadly true that the number of voluntary hospital beds throughout the country needs increasing; that the waiting list is undesirably high; and that therefore to all who understand the blessing of the voluntary system there is a patriotic as well as an interested local motive in supporting properly administered local schemes for new hospitals in England.

### DEATH OF A HOSPITAL TRUSTEE.

By the death of Mr. Russell Duckworth, writes a correspondent, the institutions of Bath lose a sympathetic and active supporter. Mr. Duckworth was the senior member of the committee of the Royal United Hospital which he joined in 1881, as well as being senior trustee of the hospital, to which position he succeeded in 1891. He had also been a governor of the Royal Mineral Water Hospital since 1878, and had served on its committee since 1879 and became one of the vice-presidents in 1896. The Bath Eye Infirmary also numbered him among its committeemen, and for thirty years Mr. Duckworth had been president of the Southern Dispensary—a position he vacated last year.

### GUARDIANS AND THE FEEBLE-MINDED.

It is interesting to note that the combination of Poor-Law Guardians for the special purpose of making provision for the care of the feeble-minded is making headway throughout the country. The Local Government Board have just issued an Order combining all the Surrey Unions for this purpose.

### THE SEX INSTRUCTION CONTROVERSY.

Lord Hill, chairman of the Elementary Education Sub-Committee of the London Education Committee, has presented a report on the question whether sex hygiene should be taught in elementary schools or not. The report was not in favour of class teaching, if only because the teachers themselves would require to be taught. Lord Hill favoured the giving of instruction and advice to the older boys and girls on leaving school by the head masters and mistresses. The discussion, in which the Rev. Dr. Scott Lidgett took part (saying that he thought there was very little connection between physiological instruction and moral conduct), strikingly illustrated the conclusions arrived at in our article on "The Facts of Syphilis on the Stage," which appeared in The Hospital of May 16. Other speakers declared that the dangers of class teaching would be far less than the dangers of no teaching at all. No one wants a hurried solution, but the expression or discussion of views is all to the good.

### THE VALUE OF THE FESTIVAL DINNER.

The summer season of hospital festival dinners has now begun; we have an aftermath also in the autumn, and, in the light of recent experience, it is worth while to consider how far the hospital banquet fulfils the claims of those who are strongly in its favour. The recent anniversary banquet of the French Hospital and Dispensary, at which a sum of £4,014 was realised, is a striking example of what may be effected on an occasion of this kind. Not only was the financial result highly satisfactory, but the whole event was thoroughly well organised and carried out by enthusiastic workers, stewards, and public officials. No doubt in the latter respect the French Hospital occupies a somewhat peculiar position. It is not every hospital, even among the narrow class of foreign institutions, which can stand to symbolise the validity of a national "entente." It is not every hospital which can secure the presence of an Ambassador, or his representative, and the Lord Mayor. In short, a good many hospital dinners pass off without being "public events" and attracting genuine public attention. But it cannot be too strongly urged that every hospital dinner can avoid degenerating into a restaurant party, and that no hospital need invite as principal speakers those titled or untitled nobodies who are really nothing more than professional diners-out. If we may take, with a grain of salt, the impression of one lay journalist who described the French dinner, we see that the very menu itself had something more—though not as much as the writer's somewhat hectic pen imagined—than mere gastronomic virtues. Brains had gone to its choosing as well as cooking; and brains are not always as conspicuous as they might be in the organisation of festival dinners. It is not enough to choose a fashionable restaurant, even to secure stewards and large subscriptions; it is little better than useless to rely on people who are chattered about in the papers as "speakers," and people who would like to be chattered about as "guests." The subscriptions must be arranged for, but the occasion itself must be made a platform from which authoritative speakers acquainted with hospital life and work can interest those present, and command the attention of the public to the voluntary system through the particular institution on whose behalf the dinner is held. Really capable and competent speakers are more important than "popular artistes," and every effort must be made to raise the occasion from a social to the level of a public function.

### A MEDICAL SUPERINTENDENT'S MISTAKE.

A patient in the Lewis Colony for Epileptics was the subject of an inquest at Warford, Cheshire, last week, at which the evidence showed that a dose of morphia had been given to him by mistake when chloral had been intended. The medical superintendent, Dr. Dougal, stated that he took full responsibility upon himself. He declared that he had in his pocket two bottles, one of which he passed over to the patient's nurse. Soon afterwards he discovered that the bottle of chloral was unopened. No sooner, he added, had the mistake been discovered than restoratives were applied, but the patient failed to respond to them and died. The verdict of "Death from misadventure was returned." It is, of course, true that no rules or regulations can completely do away with the possibility of accidents in institutions, and therefore we are tempted to leave the question at that, but at the same time it is clear that, so far as possible, a medical officer should avoid carrying dangerous drugs in his pocket. Moreover, however natural it may be on occasion to pass something over to one of the staff when it is needed, poisonous drugs should be obtainable only on requisition from the dispensary or the poison cupboard. Absence of formality leads to absence of care, and absence of formality was apparently the cause of the present mistake.

### MEDICAL STAFF APPOINTMENTS IN GLASGOW.

In view of the approaching completion and opening of the Royal Hospital for Sick Children at Yorkhill, Glasgow, the directors have made several appointments, to take effect when the move is made from the old hospital in Scott Street. Dr. T. K. Dalziel and Mr. R. H. Parry, surgeons; and Dr. R. B. Ness and Dr. J. B. M. Anderson, physicians, become respectively consulting surgeons and consulting physicians to the new hospital. Dr. A. A. Young and Dr. A. MacLennan are appointed visiting surgeons, and Dr. L. Findlay visiting physician at Yorkhill. Following the example set last year by the Western Infirmary, the managers have decided that the visiting staff shall not at the same time hold appointments to any other institution; and the recent promotions, according to the local medical papers, have been made subject to this condition.

### THE RETROGRADE POLICY OF THE BATH GUARDIANS.

We regret to see that the Bath Guardians have decided by thirty votes to twenty to postpone for one year the consideration of the question of building a new hospital for the workhouse. For some time past Dr. Curd, one of the members, has tried to convince the Board of the necessity for building a hospital of 128 beds for the sick in the workhouse. In this he was supported by Dr. Fuller, one of the medical inspectors of the Local Government Board. Both Dr. Fuller and Dr. Curd pointed out that the accommodation in the workhouse was totally unfit for the sick. It was shown that the hot-water supply and heating were inadequate, and that the sanitary arrangements and bathrooms were open to severe criticism from the hospital point of view. Worst of all, it was said that there was no provision for sick children, who had to be placed in beds between the adults, where room could be found for them, and that the workhouse infirmary was in such bad repute amongst the sick poor that they refused to enter it, saying they would rather die at home. Dr. Fuller said that the present building was such that he would not like to carry out even a minor operation within it. In face of this grave indictment by two experienced and expert medical men, it is difficult to understand how the Guardians could have come to the decision they did. The matter is one which the inhabitants of Bath, if they value the reputation of their city, cannot allow to remain in its present state. Dr. Curd has resigned his membership of the Guardians' hospital committee on the grounds that he cannot be associated with the administration of an institution which he knows to be inadequate and inefficient. We are glad to observe, however, that he intends to remain on the Board and to take every opportunity of pushing forward the claims of the sick poor.

### STAINES' NEW COTTAGE HOSPITAL.

The opening of the Staines Cottage Hospital has an interest apart from its intrinsic one—namely, that the slow wheels of discussion and delay were only hastened to a decision and a building actually erected when the late Mrs. James Harris left a legacy to the hospital on the condition that it was built within two years. A curious point also has been the decision of the Urban District Council, taken after a referendum which resulted in a majority of three to one in favour of the plan, to make good any deficiency in the upkeep of the institution for the first three years of its life. The hospital has now opened in Kingston Road, and the building, which has cost £1,250, accommodates six beds for adults and two for children. Mr. T. Leslie Moore, A.R.I.B.A., is the architect, and explained at the opening that the shape of the institution is that of a Y, the stem representing the administration and the arms the two wards. Mr. H. Scott-Freeman is the honorary secretary, the matron is Miss Bramwell, and Mr. R. Fox-Warner is the chairman of the committee.

### IMPROVEMENTS AT ST. JAMES'S INFIRMARY.

The Wandsworth Board of Guardians have sanctioned a number of improvements at St. James's Infirmary with a view to bringing it up to date. These include the provision of sunning balconies, wood and glass screens across the ends of the wards for children, additional dining-room accommodation, seven more bedrooms for the nurses, a larger patients' own clothing store, and the alteration of the present store to form a chapel for religious services. When the Guardians approved of these alterations, Mrs. Cassidy inquired if these would be the final improvements at this infirmary, and was answered by a chorus of "No." It is in the best interests of the ratepayers and patients alike that every facility should be accorded to restore the latter quickly to health. Prolonged institutional life is not beneficial to the great mass of patients who are treated in Poor-Law infirmaries. As a rule, they are too prone to take any chance of a long rest, and the longer the rest the less they like to go back to their everyday work and responsibilities.

### RELATION OF ENVIRONMENT TO WORSHIP.

In this connection let all readers note also the decision to provide a chapel for religious services. At St. James's hitherto the staff have had to worship in a committee-room, and the fact that this has been given up is only the latest proof, if proof were needed, that environment, as we recently noted, in institutional life plays an important part in relation to worship. The medical superintendent, we understand, has long been anxious for this change to be brought about.

### THE ILLNESS OF HOSPITAL SECRETARIES.

In the many statistical tables that are now published investigating the healthiness or unhealthiness of different occupations there is, so far as we are aware, no mention made of the profession of hospital secretary. Doubtless, this class is numerically too small to invite the attention of the statistician, but, without attempting to draw a general inference on this occasion, we regret to note several cases of illness among hospital secretaries at the present time. Mr. W. G. Carnt, as we recently noted, is completing his convalescence after a serious operation, and Mr. E. Lionel Blake, the secretary and superintendent of Oldham Royal Infirmary, has also been in the doctor's hands; so has Mr. J. H. Shaw, the secretary of Southport Infirmary. We are afraid that instances could also be found among the secretaries of Southern institutions. No doubt there are many questions involved in a consideration of the relative healthiness of the profession of hospital secretary which cannot be discussed here; such as age, time of year, and purely individual factors. But we suggest that to any enthusiastic statistician—say, Mr. Godfrey Hamilton, for instance—an investigation of the general healthiness of the hospital secretary would be a valuable subject for holiday or leisure research.

### THE PHARMACEUTICAL COUNCIL ELECTION.

The result of the Council election of the Pharmaceutical Society, declared at the end of last week, was that all the seven retiring members were re-elected. The three other candidates, including Mr. Herbert Skinner, whose candidature was of especial interest to institutional dispensers, were therefore unsuccessful. It is, nevertheless, satisfactory and encouraging for dispensers to know that Mr. Skinner polled a little short of fifteen hundred votes; this is more than sufficient to justify his candidature, and suggests the probability that some future attempt to secure direct representation for the class of pharmacists to which Mr. Skinner belongs will be successful. It is, by the way, a curious fact that out of the eight thousand members of the Society rather less than four thousand exercised the privilege of voting. The proportion of voters is nearly always about the same, but one would have thought that at a time when so much of interest and importance is happening to affect the calling of pharmacy the poll would have been much heavier. While dispensers will regret that their representative was not successful on this occasion, they will find consolation in the fact, which has already been pointed out in these columns, that in Mr. Edmund White, the popular president of the Society, they have a leader with a very large experience of hospital work and in Mr. Woolcock, the Society's very able secretary, a pharmacist who was for some years engaged in institutional pharmacy.

### INSURED WOMEN AND THE DRUG FUND.

In pursuance of its intention, as announced in The Hospital last week, to investigate the position of affairs in Insurance areas where there is a deficiency in the drug fund, the Pharmaceutical Society recently convened a meeting of representatives of Insurance Committees, Panel Committees, and Pharmaceutical Committees in Lancashire, at which the proposal was made that a central authority should be set up for the Insurance areas in that county for the purpose of finding out definitely whether the practice of excessive prescribing is prevalent, and if so, of setting in motion the machinery furnished by the medical benefit regulations for surcharging the doctors against whom a charge of overprescribing is proved. A committee was appointed to consider the proposal in detail and to report thereon. Another proposal is that in areas where insured women exceed in number the insured men a larger sum should be credited to the drug fund, the amount per head for women being 2s. 6d. and for men 1s. 10d. It has been the experience of Lancashire industrial districts that sickness among women is to some extent responsible for the heavy drain on the drug fund, and hence this proposal has been submitted for consideration. If it is found on investigation that the deficiency in the drug fund is not due to extravagant prescribing, it may be assumed that it is due to excessive sickness, and in the latter case it may be found desirable for the chemists to claim an extra allowance for drugs as provided by the Insurance Act.

### AMERICAN INSTITUTIONS FOR MENTAL DEFECTIVES.

On the ground that the knowledge which would be gained from such a visit would be valuable in connection with her work, the Establishment Committee of the London County Council propose to grant leave of absence, without pay, to Miss J. L. D. Fairfield, a medical assistant in the Public Health Department, for a period not exceeding two months, in order that she may visit Chicago and other American cities. The main objects of Miss Fairfield's journey are to study well-known American institutions for the mentally defective, to observe new methods of diagnosis and schemes for the care of children, and to investigate the medical department of Wellesley and other women's colleges.

### THE WELFARE OF THE BLIND: NEW PROPOSAL.

Various representations have been made by organisations interested in the welfare of the blind, and indeed officially, we understand, to the President of the Local Government Board, to the effect that representatives of the blind should be placed upon the Departmental Committee appointed to consider the condition and needs of the blind in the United Kingdom. These views have been regarded as reasonable, as a result of which a certain number of blind persons have been invited to sit upon the Committee. The number of acceptances is not yet known, as the replies have not yet been received. There can be no doubt that when any class of persons is to be made the object of certain proposals one or more of their representatives should be chosen to sit on the body which will have the decision, or, at least, the recommendations to make, and nothing is more curious than the way in which this fact is forgotten, owing to the eagerness with which voluntary bodies whose work concerns the class under consideration are apt to arrogate to themselves the sole possession of knowledge and the sole right to influence legislation.

### RADIUM FOR GLASGOW.

A short time ago, as was noted in these columns, the question of procuring a supply of radium for the use of the Glasgow hospitals was mooted. The *Glasgow Medical Journal* now gives further particulars of the scheme, from which it appears that the matter is being taken up energetically. Sir

John Stirling-Maxwell called a meeting of influential citizens a few weeks ago in the Merchants' House, and put before them the pros and cons of the purchase of radium. He said that expenditure on radium was hardly to be commended as a sound mercantile speculation, but he advocated it strongly on scientific and philanthropic grounds. He proposed that 300 milligrammes should be obtained to form a central supply for the whole city, available for the hospitals and under certain conditions for private practice. A medical committee will control the use of the radium, and complete case records will be kept. A strong executive committee was formed, consisting of Sir John Stirling-Maxwell, Bart., LL.D.; Sir Donald MacAlister, K.C.B., M.D.; Sir Matthew Arthur, Bart.; Sir G. T. Beatson, K.C.B., M.D.; Sir James Bell, Bart., LL.D.; Mr. J. D. Hedderwick; Mr. J. A. Roxburgh; Dr. J. Barlow; Dr. J. Macintyre; Dr. J. G. Tomkinson; Dr. Nigel Stark; Dr. J. M. Thom; Dr. D. J. Mackintosh, M.V.O.; Dr. D. O. McGregor; Mr. F. Soddy, M.A., F.R.S.; Mr. F. Henderson; Mr. A. Walker; and Mr. W. Gray. The committee aim at raising about £9,000, a substantial part of which has already been promised.

### NEW SECRETARY OF THE BELGRAVE HOSPITAL FOR CHILDREN.

Mr. Thomas Clapham has been appointed Secretary of the Belgrave Hospital for Children in succession to Mr. T. W. Gregg, whose promotion to the secretaryship of the Bristol General Hospital was announced in The Hospital some weeks ago. Mr. Clapham has been for three years assistant secretary to Mr. Glenton-Kerr at the Queen's Hospital for Children, Hackney Road, previous to which he was chief clerk at Hampstead General Hospital, where he went in 1906. He is to be congratulated upon his steady rise in his profession, and, like many other successful men who have exchanged the office for the institution, we have no doubt that he will find in hospital life all the advantages of competition, with, at the same time, a more inspiring goal to work for than mere profit or individual success. Hospital work is a life in which all sides of a man's nature can find expression, a life, too, in which, while there is plenty of scope for legitimate ambition, there is also a super-selfish end in view which no man of brains and energy will find other than inspiring.

### STAFF CHANGES AT BENENDEN SANATORIUM.

At the annual meeting of the National Association for the Establishment and Maintenance of Sanatoria for Workers suffering from Tuberculosis, it was reported that the medical superintendent at the Benenden Sanatorium, Kent, Dr. J. C. Smyth, had resigned on his appointment to the County of Kent as tuberculosis officer, and that Dr. W. Scarisbrick had been appointed in his stead. The assistant medical officer, Dr. G. M. Mayberry, had also resigned, and Dr. A. Leitch had been appointed to fill the vacancy. Miss E. Dane (matron) resigned to take an appointment under the Welsh National Memorial, and Miss E. Hobson had been appointed to fill her place.

### THE DISPENSERS' HALF-HOLIDAY.

The Home Secretary has expressed the opinion that doctors' dispensaries are "shops" within the meaning of the Shops Act, and that accordingly dispensers must be given a weekly half-holiday and definite intervals for meals. This opinion is, of course, not binding on the Courts, but it would no doubt carry considerable weight with any bench of magistrates before whom a doctor was summoned to appear for failing to comply with the Act. There are probably few dispensers employed by medical practitioners who do not enjoy the privilege of a weekly holiday and regular meal-times, but if the Act is going to be rigidly enforced it will be necessary in many cases to make some rearrangement in the hours of work in order to avoid the commission of technical offences. Institutional dispensers will not be affected by this decision, for since no sale is made from a hospital dispensary the dispensary is obviously not a "shop." It is understood that it is the intention of several local authorities to take steps to ensure that doctors who employ dispensers are complying with the provisions of the Act, and it is not improbable that before long the interpretation implied by the Home Secretary's statement will be tested in a police court.

### THIS WEEK'S DRUG MARKET.

The improved condition of the drug market, noted last week, has been barely maintained, and transactions have been confined to a small scale. Fluctuations in prices, however, have been rather more numerous than usual. As anticipated, English camphor refiners have raised their quotation for camphor flowers, and Japanese refined camphor is again rather dearer, with prospects of a further advance. Ergot of rye has an advancing tendency. Menthol is dull and lower. There has been little further change in the position of jalap, but a further advance in prices is considered probable. Cod-liver oil is practically unchanged, but the advanced price is well maintained; it is difficult to foresee the tendency of prices, but it may be that quotations will not vary materially for some little time. Citric acid continues to tend upwards in price, and makers of potassium citrate, sodium citrate, and iron and ammonium citrate have raised their quotations. Opium is somewhat dearer, and in some quarters it is predicted that higher prices will be asked later on; morphine also has an upward tendency, but quotations for codeine are at present unchanged, although an advance would not come as a surprise. High prices for cantharides are well maintained. The value of essence of lemon has a lower tendency. There is a fair demand for olive oil, with no indications of any decline in value in the near future.

